# Overconsumption of Omega-6 Polyunsaturated Fatty Acids (PUFAs) versus Deficiency of Omega-3 PUFAs in Modern-Day Diets: The Disturbing Factor for Their “Balanced Antagonistic Metabolic Functions” in the Human Body

**DOI:** 10.1155/2021/8848161

**Published:** 2021-03-17

**Authors:** Abeba Haile Mariamenatu, Emebet Mohammed Abdu

**Affiliations:** ^1^Department of Biotechnology, College of Natural and Computational Science, Debre Berhan University, P.O. Box 445, Debre Berhan, Ethiopia; ^2^Department of Biology, College of Natural and Computational Science, Debre Berhan University, P.O. Box 445, Debre Berhan, Ethiopia

## Abstract

Polyunsaturated fatty acids (PUFAs) contain ≥2 double-bond desaturations within the acyl chain. Omega-3 (n-3) and Omega-6 (n-6) PUFAs are the two known important families in human health and nutrition. In both Omega families, many forms of PUFAs exist: *α*-linolenic acid (ALA), eicosapentaenoic acid (EPA), and docosahexaenoic acid (DHA) from the n-3 family and linoleic acid (LA), dihomo-*γ*-linolenic acid (DGLA), and arachidonic acid (AA) from the n-6 family are the important PUFAs for human health. Omega-3 and Omega-6 PUFAs are competitively metabolized by the same set of desaturation, elongation, and oxygenase enzymes. The lipid mediators produced from their oxidative metabolism perform opposing (antagonistic) functions in the human body. Except for DGLA, n-6 PUFA-derived lipid mediators enhance inflammation, platelet aggregation, and vasoconstriction, while those of n-3 inhibit inflammation and platelet aggregation and enhance vasodilation. Overconsumption of n-6 PUFAs with low intake of n-3 PUFAs is highly associated with the pathogenesis of many modern diet-related chronic diseases. The volume of n-6 PUFAs is largely exceeding the volume of n-3PUFAs. The current n-6/n-3 ratio is 20-50/1. Due to higher ratios of n-6/n-3 in modern diets, larger quantities of LA- and AA-derived lipid mediators are produced, becoming the main causes of the formation of thrombus and atheroma, the allergic and inflammatory disorders, and the proliferation of cells, as well as the hyperactive endocannabinoid system. Therefore, in order to reduce all of these risks which are due to overconsumption of n-6 PUFAs, individuals are required to take both PUFAs in the highly recommended n-6/n-3 ratio which is 4-5/1.

## 1. Introduction

Polyunsaturated fatty acids (PUFAs) are fatty acids which contain two or more methylene-interrupted double-bond desaturations within the acyl chain [1, 2]. PUFAs contain a methyl group in one end of the molecule (termed Omega, “*ω*” or “n”) and a carboxylic acid in the other end. The nutritionally important PUFAs for human health are members of the so-called Omega-3 (or n-3) and Omega-6 (or n-6) families [3]. In both Omega families, many forms of PUFAs exists as shown in Table 1, out of which the important forms for human health are *α*-linolenic acid (ALA, C18:3n-3), eicosapentaenoic acid (EPA, C20:5n-3), and docosahexaenoic acid (DHA, C22:6n-3) from the n-3 family, whereas linoleic acid (LA, C18:2n-6), dihomo-*γ*-linolenic acid (DGLA, 20:3n-6), and arachidonic acid (AA, C20:4n-6) from the n-6 family [2, 4]. Sources of n-6 PUFAs include the common vegetable oils used in cooking (corn, safflower, sunflower, soybean, etc.), hydrogenated oils (margarine and vegetable shortening), and foods derived from livestock animals and poultry raised on grain rather than on green pasture. On the other hand, n-3 PUFAs are sourced from green vegetables, wild ocean fish, oily seeds (chia, flax, and perilla), and foods derived from animals raised on green pasture [5–7].

Apart from enhancing *β*-oxidation and increasing the fluidity of the cellular membrane, the prominent metabolic function of n-3 and n-6 PUFAs is in signal transmission which mostly requires the release of PUFAs from the membrane by phospholipases. PUFAs regulate gene expression directly or through their derived secondary messenger substances (lipid mediators): octadecadienoids (C_18_), eicosanoids (C_20_), and docosanoids (C_22_) in the cytoplasm [6, 8–10]. Long-chain PUFAs such as AA, EPA, and DHA detach from membrane phospholipids and undergo oxidation into their respective lipid mediators using the oxygenase enzymes: cyclooxygenase (COX), lipooxygenases (LOX), and cytochrome P450 (CYP) [11, 12]. LA also has been shown as a target of COX, LOX, and myeloperoxidase (MPO) enzymes to produce other octadecadienoid (C_18_) lipid mediators having a metabolic function in inflammation [13, 14]. Thereby, these PUFAs in both families regulate inflammation, immunity, blood vessels, platelets, synaptic plasticity, cellular growth, pain, sleep, etc. Especially for inflammation, immunity, blood vessels, and platelets, derivatives of n-6 and n-3 PUFAs regulate mostly in an opposing (antagonistic) manner. Generally, n-6 enhances inflammation, platelet aggregation, and vasoconstriction, whereas n-3 inhibits inflammation and platelet aggregation and enhances vasodilation [6].

Ideally, even though these PUFAs in both families are essential to the human body, consuming higher n-6 than n-3, as is now mostly happening in modern westernized diet styles, has been shown to exert an effect on human health and in fact contribute to the emergence of many modern chronic inflammatory diseases, cardiovascular diseases, and some cancers, providing that inflammation is at the base of most of the modern diet-related chronic diseases. Derivatives of n-6 PUFAs are mostly involved in the proinflammatory reaction, unlike that of the anti-inflammatory and resolving reaction performed by n-3 PUFAs derivatives [7, 15–19]. Therefore, n-3 and n-6 PUFAs have a crucial role in determining the well-being of individuals as the lower intake (in the case of ALA, EPA, and DHA) and overconsumption (in the case of LA and AA) are now highly associated with the pathogenesis of many inflammation-induced modern chronic diseases [20–22]. In the past three decades, in modern society which is shown to follow westernized diet styles, the total fat and saturated fat intake as a percentage of total calories has continuously decreased, while the intake of n-6 increased and that of n-3 PUFAs decreased, resulting in a large increase in the n-6/n-3 ratio from 1-2/1 during evolution to 20-50/1 today [16, 18].

Due to higher ratios of n-6/n-3 in modern diets, the LA-derived proinflammatory octadecadienoids [13, 14], AA-derived eicosanoids [5, 23], and endocannabinoids [16, 19] are formed in larger quantities than those derived from n-3 PUFAs (ALA, EPA, and DHA) which have anti-inflammatory and inflammation-resolving characteristics. The large quantities of LA and AA derivatives contribute to the formation of thrombus and atheroma; to the allergic and inflammatory disorders, particularly in susceptible people; to the proliferation of cells; and to the very hyperactive endocannabinoid system (in which appetite and food intake which can lead to weight gain/obesity are increased). Thus, diets which are rich in n-6 PUFAs shift the normal physiologic state to one that is prothrombotic and proaggregatory, with an increase in blood viscosity, vasospasm, and vasoconstriction and a decrease in bleeding time and energy imbalances [16, 19, 24]. Therefore, maintaining a balanced ratio of these two PUFAs families in our diet and our daily intake is becoming very crucial with respect to the healthy development of the human body and function as well as the prevention of the emergence of modern chronic diseases. Certainly, their balance is important in decreasing the risk of coronary heart disease, hypertension, cancer, type 2 diabetes, arthritis, and other autoimmune and possibly neurodegenerative diseases [25]. The objective of this review is to show the current status of the n-6/n-3 ratio imbalances in modern diets and their effect on the metabolic function of these fatty acid groups with relation to human health.

## 2. Methodology of Literature Searching

A literature search was done for the publications until May 30, 2020, from four databases: PubMed, Scopus, Web of Science, and Google Scholar. The search terms used were as follows: Omega-3 or Omega-6 or Omega-3 PUFAs or Omega-6 PUFAs or n-3 PUFAs or n-6 PUFAs or Polyunsaturated Fatty Acids or Long chain Poly unsaturated fatty acids or Essential fatty acids or Omega-3 to Omega-6 ratio or Omega-3 dietary sources or Omega-6 dietary sources or Biosynthesis of Omega-3 or Linoleic acid (LA) or *α*-Linolenic acid (ALA) or Docosahexaenoic acid (DHA) or Eicosapentaenoic acid (EPA) or Biosynthesis of Omega-6 PUFAs or Omega-3 and Omega-6 metabolism or Omega-3 to 6 ratio and inflammatory diseases or Omega-3 to 6 ratio and chronic diseases. Publications which were cited in the retrieved articles in the first search were also selected manually based on their relevance to the topic. Articles which are only in the English language and have relevance to the topic are included in this review, whereas publications which are not in the English language and have less relevance for the topic are excluded from this review.

## 3. General Biochemistry Aspect of n-3 and n-6 PUFAs

**Figure 1:**
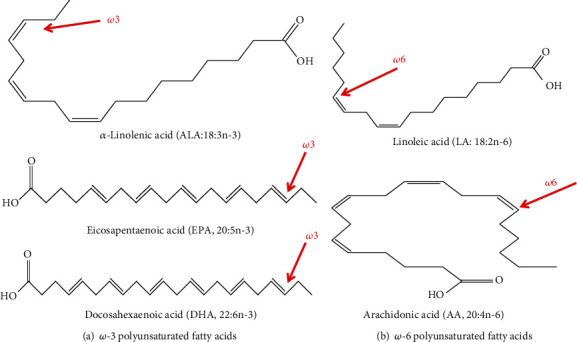
The most nutritionally important n-3 and n-6 PUFAs and their chemical structure. (a) n-3 PUFAs. (b) n-6 PUFAs. Adapted from Saini and Keum [12].

**Figure 2:**
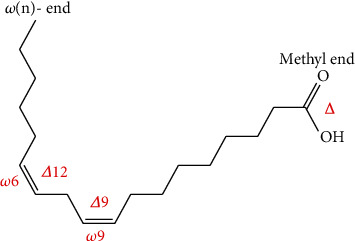
The chemical structure of linoleic acid (LA, 18:2n-6 or 18:2^*Δ*9,12^) and its two alternative systematic nomenclature. LA contains 2 double bonds, or desaturations, at the *Δ*9 and *Δ*12 carbon positions from the carboxyl (*Δ* terminus) or at the n-6 and n-9 carbon positions counted from the methyl (-n) end [27].

Fatty acids are generally hydrocarbon chains with a carboxylic group at one end and a methyl group at the other end. In fact, the biological reactivity of fatty acid is determined by the length of the carbon chain and the number and position of any double bonds present on it. According to the degree of unsaturation, the most common dietary fatty acids have been divided into three broad classes: saturated fatty acids (SFAs, have no double bond), monounsaturated fatty acids (MUFAs, have one double bond), and polyunsaturated fatty acids (PUFAs, have two or more double-bond desaturations) within the acyl chain [1, 2, 12]. While the short-chain polyunsaturated fatty acids (SC-PUFAs) have 19 or fewer carbon atoms, the long-chain polyunsaturated fatty acids (LC-PUFAs) have ≥20 carbons in their length [2, 3, 26, 27]. The double-bond desaturations of naturally occurring unsaturated fatty acids are methylene-interrupted in the *cis*-orientation (or) configurations [28], which means that the hydrogen atoms attached to the double bond are on the same side. Natural PUFAs with methylene-interrupted double bonds all in *cis*-configurations can be divided into 12 families ranging from double bonds located at the n-1 position to the n-12 position [2]. They have a methyl group in one end of the molecule (termed Omega, “*ω*” or “n”) and a carboxylic acid in the other end. The letter “n” is often used instead of the Greek “*ω*” to describe the methyl end, while “*Δ*” (Delta) is used to describe the carboxylic end [29]. In terms of the extent of occurrence and human health and nutrition, the n-6 and n-3 families are the most important natural PUFAs [2]. The nutritionally important PUFAs are grouped into Omega-3 (or n-3) and Omega-6 (or n-6) categories based on the position of the first double bond from the methyl, or Omega, fatty acid terminus (Figure 1). The term “Omega-3” or “Omega-6” refers to the position of the first double bond in the carbon chain as counted from the methyl end of the fatty acid. All n-3 and n-6 PUFAs have this double bond on carbon numbers 3 and 6, respectively, by counting the methyl carbon as carbon-1 [2, 26–28, 30]. Furthermore, it is sometimes common to use the systematic nomenclature for PUFAs which indicates the location of double bonds with reference to the first carbon in the carboxylate group as shown in Figure 2 [27, 29] and is represented by the symbol *Δ*, for example, 18:2^*Δ*9,12^ for linoleic acid (LA, C18:2n-6).

**Figure 3:**
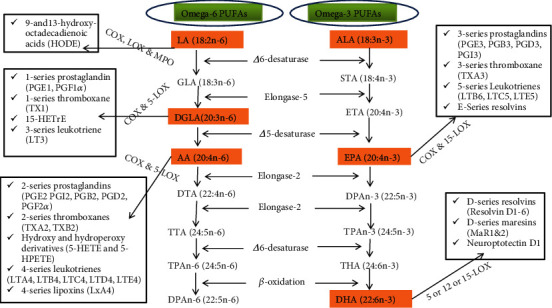
Biosynthesis of Omega-3 and Omega-6 PUFAs is shown at the center. The oxidative metabolism of LA, DGLA, AA, EPA, and DHA into their respective lipid mediators through oxygenase enzymes is also shown in the right and left sides. COX: cyclooxygenase; LOX: lipoxygenase; MPO: myeloperoxidase [19, 33, 48, 60].

The members of the two families with their systematic nomenclature are listed in Table 1. The two short-chain PUFAs, ALA (18:3n-3) and LA (18:2n-6), and the longer PUFAs such as EPA (20:5n-3), DGLA (20:3n-6), AA (20:4n-6), and DHA (22:6n-3) are the most important forms of PUFAs for human health and nutrition [2, 4]. LA and ALA are the two “essential PUFAs” in which humans must obtain from their daily diet [16, 31] as the human body cannot synthesize them for a genetic reason [32]. On the contrary, LC-PUFAs such as EPA, DHA, and AA are considered “conditionally essential” PUFAs as they have a special function in the body. Though it is in a minimized efficiency, EPA, DHA, and AA can be synthesized in the human body. For conditionally essential PUFAs, humans are encouraged to take additional amounts in such a way that it ensures the optimum daily intake of total n-3 and n-6 PUFAs according to the health status of the individual [16, 31]. LA and ALA are used as a precursor during the biosynthesis path of n-3 and n-6 LC-PUFAs [4, 33], in which they are competitively metabolized in two distinct pathways using common enzymes [7] as shown in Figure 3. Mammalian cells, including humans, cannot convert n-6 to n-3 PUFAs because they lack the converting enzymes, n-3-desaturases [32]. These two families are therefore noninterconvertible and metabolically and functionally distinct and often have important opposing metabolic functions in the physiology of the human body. While proteins in a cell are genetically determined, the PUFA composition of the cell membrane is to a great extent dependent on dietary intake. Clearly speaking, when humans take diets containing more EPA and DHA, for instance, the AA in the cell membrane of probably all cells, especially in the membranes of platelets, erythrocytes, neutrophils, monocytes, and liver cells, is partially replaced by these PUFAs [5].

In the human body, after digestion, the destruction of the triacylglycerol (TAG) structure and release of PUFAs led to the absorption and transportation in the bloodstream to the tissues where they are incorporated in their structure. PUFAs can take three different metabolic pathways. (1) They contribute to ATP production by the *β*-oxidation pathway. (2) They undergo modification (esterification) into cellular lipids as neutral (such as TAG and cholesterol ester) and polar lipids (such as phospholipids and sphingolipids). (3) They are a precursor for the process of elongation and desaturation through enzymatic reactions to create the next long-chain and more desaturated PUFAs or retroconversion to generate different metabolites [34, 35]. Generally, n-3 and n-6 PUFAs contribute to the following three physiologic functions in the body. (1) The increase *β*-oxidation. PUFAs in TAG, in addition to serving as a source of fuel, regulate transcription factors, thereby improving fatty acid oxidation. (2) They are part of the cellular membrane so that they set up and modulate it, thereby increasing its fluidity. Through their fluidity, they optimize the position, amount, and function of membrane proteins. (3) They are involved in signal transmission. Signal transmission requires the release of PUFAs from the membrane by phospholipases. PUFAs regulate gene expression directly or through their derived lipid mediator substances such as octadecadienoids (C_18_), eicosanoids (C_20_), and docosanoids (C_22_) in the cytoplasm [6, 8–10].

## 4. *De Novo* Synthesis and Accumulation of n-3 and n-6 PUFAs in Different Organisms of the Marine and Terrestrial Ecosystems

Organisms, especially almost all plants and algae, some fungi, and lower animals (such as *Caenorhabditis elegans*) possess desaturase enzymes to convert oleic acid (OA, 18:1n-9 or 18:1^*Δ*9C^) and monounsaturated fatty acid (MUFA) into LA (18:2^*Δ*9,12^) and then into ALA (18:3^*Δ*9,12,15^) [12]. *Δ*12-desaturase introduces a double bond at the 12^th^ C position of OA to form LA, while the n-3-desaturase (*Δ*15-desaturase) inserts a double bond at the 15^th^ C position of LA to form ALA. Plants can therefore synthesize these SC-PUFAs from OA by introducing desaturations onto *Δ*^12^C- for LA using *Δ*12-desaturase. The n-3-desaturases (*Δ*15-desaturases) act on the *Δ*^15^C- of LA (n-6) and produce ALA (n-3). However, plants lack the *Δ*6-desaturase activities which elongate and desaturate further these precursors into their respective longer LC-PUFAs; hence, higher plants are devoid of n-3 and n-6 LC-PUFAs [8, 12, 36, 37]. On the contrary, as it is reviewed in [8], all animals including human beings did not have the capacity to synthesize endogenously the essential SC-PUFAs, but with limited ability, they can synthesize LC-PUFAs from precursor PUFAs, LA and ALA. They lack two critical desaturation enzymes: *Δ*12-desaturase and *Δ*15-desaturase [32]. For most animals including humans, therefore, it is a must to obtain these essential short-chain fatty acids from their dietary intake. In the animal body, these SC-PUFAs can be metabolized into more bioactive and longer PUFAs through a series of steps of desaturation and elongation reactions as shown in Figure 3 [36]. Following these reactions, LA and ALA can be converted into their respective longer and more desaturated PUFAs, though in the latter, the conversion efficiency is very limited [37]. Due to this limited conversion capacity, humans are encouraged to take n-3 PUFAs such as EPA and DHA supplements [2, 38].

A huge majority of n-3 and n-6 PUFAs especially LC-PUFAs are produced *de novo* at the base of the marine food web in marine microbes, predominantly microalgae such as *Crypthecodinium cohnii*, *Schizochytrium*, and *Nannochloropsis* [39]. In these microorganisms, n-3 and n-6 PUFAs are synthesized *de novo* by the aerobic pathway, which is well discussed in [36, 37, 40, 41]. Microalgae, therefore, are the primary *de novo* producers of nutritionally important PUFAs in the aquatic environment, providing a continual supply of PUFAs like AA, EPA, and DHA by concentrating up the tropic food chain, such as in fish, where there is limited capacity to synthesize these beneficial PUFAs. Clearly, these fatty acids accumulate in the lipids of marine fish such as mackerel, salmon, herring, trout, sardines, and tuna [35, 39, 42] and other invertebrates like the Antarctic and Pacific krill (small shrimp-like crustaceans) [43, 44] and *Calanoid copepods* [45] feeding on microalgae. Fish, which are the main dietary sources of LC-PUFAs especially n-3, are inefficient at converting ALA to n-3 longer PUFAs at a considerable level of the beneficial EPA and DHA like that of human beings. Hence, they must be getting them from their respective marine feed like microalgae and smaller invertebrates [46]. Therefore, the marine ecosystem is characterized by the high level of longer chains of PUFAs like EPA, DHA, and AA that originate in microalgae (phytoplankton) and accumulate up the food chain in fish and some zooplankton, whereas the terrestrial ecosystem is known by the high level of short-chain PUFAs especially ALA and LA mainly produced *de novo* in different plants, oilseed crops and vegetables.

## 5. Typical and Usual Dietary Sources of n-3 and n-6 PUFAs

Naturally, LA is abundant and found in seeds (grape, rape, poppy, hemp, and almond), nuts (Brazil nuts, walnuts, pine nuts, and hazelnuts), and vegetable oils (sunflower, safflower, corn, cottonseed, soybean, coconut, and palm). These sources contain a significant amount of LA with a low proportion of ALA as shown in Table 2. On the other hand, as indicated in Abedi and Sahari [37], ALA is found in a relatively higher amount in few oily seeds (chia, perilla, flax, basil, and *Camelina*). ALA is also found in the chloroplast of cultivated green leafy vegetables (cauliflower, broccoli, kale, and lettuce), seeds and nuts (walnut, beechnut, soybean, rapeseed (canola), pumpkin, red currant, and black currant), and fruits (avocados, raspberries, and strawberries) [6, 7, 19]. Green leafy vegetables contain high proportions (60-70% of total FAs) of PUFAs in the form of ALA [12]. As it is well reviewed in Abedi and Sahari [37], ALA is also found in many wild edible plants (such as *Verbena officinalis* L. (vervain), *Chenopodium album* L. (goosefoot), *Picris echioides* L. (oxtongue), and *Sonchus oleraceus* L. (sowthistle)) in much considerable amounts (43.20-54.99% of ALA). From the derivative of n-6, GLA, which is typically consumed as part of dietary supplementation, is found in human milk and some botanical seed oils such as evening primrose oil (EPO), borage oil, and black currant oil [35, 37, 47]. AA is found predominantly in the phospholipids of grain-fed animal and poultry products such as meat, lard, turkey fat, butter, and egg lipids. EPA and DHA are mostly found in breast milk, oily marine fish, and fish oils (such as salmon, mackerel, sardines, anchovies, herring, and rainbow trout) as well as microalgal oils, squid oils, and krill oils [2, 5–7, 27, 35, 47, 48]. Wild marine fish and krill are potential sources for n-3 PUFAs, since the fish feed on phytoplankton (mostly the microalgae) and other zooplankton (small fish and invertebrates) while the krill feed on microalgae, the primary producers of these PUFAs [12].

Currently, in today's world, the most common human dietary intake of essential PUFAs contains mainly LA and ALA, but LA is much prevalent than ALA. Though humans could get ALA from dietary oilseed crops and vegetables, the conversion efficiency to n-3 longer PUFAs is still limited [12]. On top of this, the natural sources we used as our food today are mainly sourced from land plants, vegetables, and animals and are not rich in n-3 LC-PUFAs like EPA and DHA, unlike the marine-based sources. The marine-based sources (fish and fish oils, microalgal oil, krill oil, and squid oil) are not equally easily accessible and affordable and are not in enough amounts for all human beings [6, 12]. Therefore, it is not easy to get n-3 LC-PUFAs especially EPA and DHA in most modern diets as well as to biosynthesize in enough amounts from dietary ALA precursors like that of n-6 LC-PUFA counterparts. More than 98% of PUFAs in plant- and animal-based foods are found in the form of TAGs, followed by PLs and diacylglycerols (DAGs), cholesterol ester (CE), and fat-soluble vitamin esters. PUFAs found in these forms have shown significantly different levels of bioavailability in animals and humans. PUFAs in the form of PLs are more bioavailable due to PLs' superior water dispersing ability and greater susceptibility to phospholipases compared to TAGs [12, 49]. But in some lower animals like krill, the percentage is different. Krill oil contains more efficient (bioavailable) DHA (35% of DHA found in the form of PLs) than fish oil (which is mostly found in the form of TAGs). Since the brain has higher uptake of PLs, PUFA supplementations in the form of PLs such as krill oils are more efficient than PUFAs in TAGs [12, 50].

## 6. The n-6/n-3 Ratio Imbalances in Modern Westernized Diets

As it is described in Simopoulos [24, 52], it was over 10,000 years ago (i.e., since the beginning of the agricultural revolution) that the overall human diet, including energy intake and expenditure, has changed. After the onset of the industrial revolution (150-160 years back), however, major changes in the human diet, particularly in the type and amount of both the essential and conditionally essential fatty acids, have taken place. Today, industrialized societies which follow westernized diet styles are characterized by an increase in overall energy intake, n-6 PUFAs, and cereal grains, whereas a decrease in energy expenditure as well as a decrease in the intake of n-3 PUFAs, complex carbohydrates and fiber, fruits and vegetables, protein, antioxidants, calcium, and vitamin D [53].

The ratio between n-6 and n-3 has been widely researched, and it was believed that our ancestors in the Paleolithic period (evolutionary times) consumed n-6 and n-3 in a ratio of 1-2/1, which is believed as a perfect and balanced ratio [5, 24, 52, 54]. Over the progression of human evolution, however, there has been a gradual change in the evolutionary norms of the n-6 to n-3 PUFA ratio consumed in the diet while a radical change has been observed following the onset of the industrial revolution [5, 16, 54]. As it is indicated in Chaves et al. [18], between 1935 and 1939, the ratio of n-6/n-3 was 8.4/1. In 1985, this ratio was 10.3/1 and even as high as 12.4/1 in other calculations. Later, between 2001 and 2011, the average ratio of n-6/n-3 was reported as 15-16.7/1 [5, 24, 25, 47, 52, 53, 55, 56]. Today, in western diet styles, the estimated average ratio is raised to 20/1 [7, 16], with a ratio as high as 50/1 in South Asia [18]. In the past three decades, in the diets of modern society, total fat and saturated fat intake as a percentage of total calories has been continuously decreased, while the intake of n-6 PUFAs increased and that of n-3 decreased, resulting in a large increase in the n-6/n-3 ratio and becoming 20-50 times the evolutionary times. This change in the n-6/n-3 ratio, possibly more than any other dietary factor, has contributed to the significant increase in the prevalence of body tissue and systemic inflammation and overweight/obesity which altogether leads to an epidemic of other diet-related chronic noncommunicable diseases such as coronary heart disease, hypertension, cancer, type 2 diabetes, arthritis, and other autoimmune and possibly neurodegenerative diseases [18].

While the most optimal homeostatic level of n-6/n-3 ratios that must be in our body or cellular concentration is 1-5/1 [33, 56, 57], there are many obstacles that hinder in maintaining this ratio between the two families, out of which the following two are the basic ones: (1) metabolic competition of ALA and LA for the same set of enzymes and (2) the prevalence of westernized diet styles in modern society [58]. Modern westernized diets, unlike that of Mediterranean and Indo-Mediterranean diet styles, contain too much n-6 but a lower level of n-3 in the form of both SC-PUFAs and LC-PUFAs. Mediterranean and Indo-Mediterranean diet styles are rich in n-3 PUFAs with a well-balanced ratio of n-6/n-3, i.e., approximately similar to the Paleolithic diet [7, 35]. A higher proportion of n-6 PUFAs in westernized diets is possibly due to the following three general reasons: (1) the emergence of modern agriculture and agribusiness along with processed food, (2) the advent and development of modern vegetable oil industries focusing on hydrogenation and refinement of vegetable oils, and (3) the recommendation of different agencies and authorities to eat fats and oils made of unsaturated fatty acids than saturated fats in order to prevent cardiovascular diseases [12, 18, 54, 59].

In modern agriculture, the emphasis is on production, i.e., on food security rather than functional food security. Unfortunately, this contributed to food security with western diets that are rich in n-6 PUFAs while the n-3 PUFAs content in many foods has been decreased [18]. As it is discussed in Simopoulos [16], the cultivation system (culture) in modern agriculture had to limit the option of edible wild plant foods having a high level of n-3 PUFAs as well as a balanced ratio of n-6/n-3. Some cultivated plant species have been shown to contain less n-3 PUFAs than their relative wild (noncultivated) edible species. Edible wild plants contain very good amounts of n-3 PUFAs in the form of ALA than cultivated related plants, as well as a balanced n-6/n-3 ratio [16, 37]. The use of cereal grains devoid of n-3 PUFAs as animal feed for domestic livestock has increased which in turn altered the PUFA profile of the food produced from them such as animal meats, eggs, and even fish. Traditionally, animals which were raised on freely grazing grass contain ALA, whereas grains such as corn and soybean, the main feed of farm animals, are high in LA [15]. Modern aquaculture produces fish that contain less n-3 PUFAs than do fish grown naturally in the oceans, rivers, and lakes. The PUFA composition of egg yolk from free-ranging chicken has an n-6/n-3 ratio of 1.3/1 whereas the USDA egg has a ratio of 19.9/1. However, the ratio of n-6/n-3 PUFAs in chickens reared on feed which is enriched with fish meal or flaxseed, decreased to 6.6/1 and 1.6/1, respectively. The vegetable oil industries mostly use oilseed crops and vegetables such as corn, sunflower, safflower, cottonseed, and soybean which contain much of LA than ALA as a raw material [25]. For the last three decades, following the recommendation of different agencies and authorities to eat unsaturated fatty acids than saturated fatty acids, intake of fatty acids in modern society has been shifted from saturated fatty acids (butter, lard) to PUFA-containing vegetable oils rich in the n-6 LA and poor in the n-3 ALA and has a higher ratio of n-6/n-3 [51, 54] when compared to fish [51] and microalgal oils [48] as shown in Table 3. This shift is probably one of the reasons that led to a remarkably significant increase in the ratio of n-6/n-3. Omega-6 PUFAs have been accumulated in the body at the expense of n-3 PUFAs. Such diets having much higher n-6 PUFAs have been adopted by an increased number of populations in the western world as well as in the urban populations of middle-income countries [18].

## 7. Competitive Metabolism of n-3 and n-6 PUFAs in the Human Body

### 7.1. LA and ALA Conversion to Longer and More Desaturated PUFAs

In humans, the metabolism of LA and ALA to other longer chains, more unsaturated n-3 and n-6, occurs by a series of linked desaturation and elongation reactions that mainly takes place in the liver and is then delivered to other cells [12, 60]. While elongation and desaturation reactions transform essential short-chain fatty acids into LC-PUFAs and more unsaturated derivatives of PUFAs, retroconversion (shortening of the chain) resulted in a shortening of two carbon units of the longest PUFAs as in the case of DHA production using *β*-oxidation from THA (24:6n-3) [34]. When n-3 and n-6 PUFAs are consumed, they compete for incorporation into cell membranes in all tissues of the body. In the synthesis of longer PUFAs (such as AA, EPA, and DHA), ALA and LA strive for the same metabolic pathway which uses the same desaturation enzyme, *Δ*6-desaturase. It has been observed that too high an intake of LA would reduce the level of *Δ*6-desaturase available for the metabolism of ALA [7, 61]. Hence, a higher intake of ALA results in the increased production of anti-inflammatory eicosanoids and other autacoids due to increased synthesis of EPA and DHA. LA can be converted to ALA in the majority of higher plants, algae, and fungi because they possess *Δ*12- and *Δ*15-desaturases but not in the human body and other mammals [12, 37].

Starting from LA and ALA, but following two distinct series of linked desaturation and elongation pathways, many LC-PUFAs are produced enzymatically step by step from respective precursors as shown in Figure 3. This enzymatic cascade produces many derivatives, among which DGLA, AA, EPA, and DHA are the most prominent PUFAs with respect to human health and development. In n-6 families, DGLA is produced from LA after one round of desaturation and elongation. DGLA is the precursor of series-1 prostaglandins (PG1) as well as to AA. *Δ*5-desaturase can convert DGLA to AA, a precursor of series-2 prostaglandins (PG2) and thromboxanes and series-4 leukotrienes. In n-3 families, EPA is produced after one round of desaturation (by *Δ*6-desaturase), one elongation (by elongase-5), and lastly one desaturation (by *Δ*5-desaturase). EPA is also a precursor of series-3 prostaglandins (PG3) and series-5 leukotriene (LT). In mammals, from AA and EPA, two cycles of elongation (by elongase-2) and one desaturation (by *Δ*6-desaturase) take place to form TPA_n-6_ (24:5n-6) and THA (24:6n-3), respectively. Then, these two PUFAs are transferred from the endoplasmic reticulum (ER) to peroxisomes, the so-called Sprecher's shunt, where they undergo limited *β*-oxidation to generate DHA (22:6n-3) and DPA_n-6_ (22:5n-6) which both return to the ER [7, 33, 35, 37]. Biosynthesized PUFAs (DGLA, AA, EPA, and DHA) are stored in the esterified form in PLs or as neutral glycerides and can be mobilized later when needed by phospholipase A2 as free (unesterified) PUFAs to form potent eicosanoids such as prostaglandins (PGs), thromboxanes (TXs), leukotrienes (LTs), lipoxins (Lx), and hydroxyeicosatetraenoic acids (HETEs) and other autacoids such as protectins, D-series resolvins, and maresins by the set of oxygenase enzymes: cyclooxygenases (COX-1 and COX-2), lipoxygenases (5-LOX, 12-LOX, and 15-LOX), and epoxygenase (cytochrome P450 or CYP) [12, 35].

### 7.2. Oxidative Metabolism of n-3 and n-6 PUFAs into Potent Lipid Mediators and Their Opposing Metabolic Functions

Omega-3 and Omega-6 PUFAs play an important role in the composition of all cell membrane PLs where they maintain homeostasis for correct membrane protein function and influence membrane fluidity and regulation of the cell signaling process, cellular function, and gene expression [6, 8–10]. Though both n-3 PUFAs and n-6 PUFAs are generally considered to have beneficial health effect, they have antagonistic (opposing) effects on metabolic functions in the body which later may lead to the emergence of many of the diet-related pathological processes in the human body if the balance of “antagonistic metabolic functions” is altered [12]. Members of the n-3 and n-6 PUFA families exert opposing metabolic functions having a significant role in regulating body homeostasis of proinflammation and anti-inflammation, vasodilation and vasoconstriction, bronchoconstriction and bronchodilation, and platelet aggregation and antiaggregation [62]. These PUFAs may exert effects by themselves to regulate diverse sets of homeostatic processes or by locally acting bioactive signaling lipids called octadecadienoids [13, 14], eicosanoids, and docosanoids (autacoids) [63].

While eicosanoids are the oxygenated derivatives of C_20_ PUFAs such as AA, DGLA, and EPA, with proinflammatory or anti-inflammatory characteristics, autacoids are derived from C_22_ PUFAs (mostly DHA) and have neuroprotectins and inflammation-resolving characteristics [12]. Recently, the oxidized derivatives of C_18_ n-6 PUFAs (especially LA-derived octadecadienoids) also show proinflammatory effects [13, 14]. Eicosanoids and autacoids are biologically active lipids which all have been implicated in various pathological processes such as inflammation and cancer [60, 64]. Especially the eicosanoids, when they are synthesized in high quantities, they influence various metabolic activities: activation of leukocytes, platelet aggregation, regulation of gastric secretions, bleeding, induction of vasoconstriction, vasodilation, bronchoconstriction, bronchodilation, and signaling of pain in nerve cells; thereby, they regulate inflammation, immunity, blood vessels, platelets, synaptic plasticity, cellular growth, pain, sleep, etc. [60, 65].

The metabolic functions performed by PUFAs mostly require detachment of LC-PUFAs from membrane PLs and oxidative metabolism into their respective eicosanoid or docosanoid derivatives [11, 12]. When cells are activated by external stimuli, like the binding of growth factors and hormones to the cell membrane receptors, phospholipase A2 is activated and releases free PUFAs mainly DGLA, AA, EPA, and DHA from *β* (sn-2 position) of PLs. These free PUFAs undergo oxidation and produce different eicosanoids and docosanoids (autacoids) using COX and LOX enzymes [12] (Figure 3). The oxidation of C_20_ eicosanoic acids (AA, DGLA, and EPA) to prostaglandins and thromboxanes, which are collectively known as prostanoids [60], is catalyzed by COX. COX converts DGLA to PG1, AA to PG2, and EPA to PG3 endoperoxides. Further metabolism of these endoperoxides to characteristic products depends on the cell type. For example, in arterial endothelial cells, PG2 is largely converted to PGI2 (prostacyclin), which causes vessel vasodilation and also inhibits platelet aggregation. In platelets, however, PG2 is converted to TXA2 which is important to normal blood clotting, since it causes vasoconstriction and platelet aggregation. These metabolites must be continuously synthesized as they have a very short half-life. These various products tend to act in a “balanced antagonistic” way. While TXA2 is the most potent proaggregatory agent known, it is normally counteracted by prostacyclin. When the balance is altered, excess thromboxane leads to abnormal platelet adhesion and aggregation, and therefore, it may be implicated in coronary thrombosis [31].

The activity of COX enzymes on AA leads to the synthesis of series-2 prostaglandins (prostaglandin E2, prostaglandin I2 (prostacyclin I2), and thromboxane A2), while the activity of 5-LOX metabolizes AA to hydroxyl and hydroperoxy derivatives: 5-HETE and 5-hydroperoxyeicosatraenoic acid (5-HPETE). These derivatives in turn are used to produce series-4 leukotrienes: LTA4, LTB4, LTC4, LTD4, and LTE4 according to the cell type such as monocytes, macrophages, neutrophils, mast cells, eosinophils, and basophils [60]. The synthesis of AA-derived eicosanoids is influenced by the concentration of DGLA. When DGLA is in excess, it competes with AA for COX and LOX so that inhibiting the production of AA-derived eicosanoids and driving the synthesis of series-1 prostaglandins (especially PGE1) and 15-hydroxyeicosatrienoic acid (15-HETrE) as DGLA lead to a higher affinity to COX and LOX enzymes [60, 66]. Similar to AA, EPA and DHA are also converted by the same set of enzymes, COX and LOX. EPA is metabolized by COX and LOX enzymes to series-3 prostaglandins (prostaglandin E3, prostaglandin I3 (prostacyclin I3), and thromboxane A3) and series-5 leukotrienes (B5, C5, and D6), respectively, with their potent anti-inflammatory, vasodilator, and antiaggregatory functions. LOX also acted on DHA and converted it into autacoids such as protectins, D-series resolvins, and D-series maresins [12, 35] as shown in Figure 3. Series-1, series-2, series-3, series-4, and series-5 refer to the number of double bonds found in each derivative (e.g., series-1, one double bond; series-2, 2 double bonds).

In addition to the *Δ*6- and *Δ*5-desaturase enzymes, the PUFAs in n-6 and n-3 families compete for the oxygenase enzymes (COX and LOX). The higher concentration of EPA and DHA in membrane PLs reduces the amount of eicosanoid production from AA just by competing for the same sets of enzymes [55]. Therefore, competition between the n-6 and n-3 PUFAs also occurs in the synthesis of prostaglandin, thromboxane, and leukotriene and hydroxyl and hydroperoxy derivatives. EPA competes with AA for prostaglandin, thromboxane, and leukotriene synthesis at the COX and LOX levels. As it is well discussed in the same author, when humans take diets rich in n-3 such as EPA and DHA, the following metabolic changes are taking place in the body: (1) a decreased production of prostaglandin E2 (PGE2) metabolites; (2) a decrease in thromboxane A2, a potent platelet aggregator and vasoconstrictor; (3) a decrease in leukotriene B4 formation, an inducer of inflammation and powerful inducer of leukocyte chemotaxis and adherence; (4) an increase in thromboxane A3, a weak platelet aggregator and weak vasoconstrictor; (5) an increase in prostacyclin PGI3, leading to an overall increase in total prostacyclin by increasing PGI3 without a decrease in PGI2, where both PGI2 and PGI3 are active vasodilators and inhibitors of platelet aggregation; and (6) an increase in leukotriene B5, a weak inducer of inflammation and a weak chemotactic agent [55].

Although AA is the known proinflammatory Omega-6 PUFA, in a recent study done on an inflammatory model *Drosophila*, it has been indicated that LA enhances inflammation by the production of a nonprostaglandin lipid mediator, 9-hydroxy-octadecadienoic acid (HODE), while ALA has been shown to significantly suppress the incidence of inflammation [14]. In fact, in a former study done by Kubala et al. [13], in mammals including humans, LA has been shown to produce 9- and 13-HODE enzymatically due to the activity of COX, LOX, and myeloperoxidase (MPO) in addition to free radical-triggered oxidation, with 9-HODE being elevated during inflammation. So taking these two together, the increased inflammation caused by increased intake of n-6 PUFA may also be due to the LA-derived 9-HODE. In general, we can say that n-6 PUFA-derived octadecadienoids and eicosanoids are proinflammatory, whereas n-3 PUFA-derived octadecadienoids, eicosanoids, and docosanoids (autacoids) have anti-inflammatory and even resolving and protecting roles. The ratio of the n-6/n-3 PUFAs in the diet determines the level of production of proinflammatory or anti-inflammatory lipid mediators from the oxidation. If the level of n-6 PUFAs in PLs is much higher than that of n-3, more proinflammatory lipid mediators are produced. These proinflammatory and anti-inflammatory octadecadienoids and eicosanoids regulate homeostatic and inflammatory processes connected with infection, inflammation, and cancer formation [63].

Though acute inflammatory response can protect the host against infection and injury [67], uncontrolled and inappropriately activated acute inflammation due to excess inflammatory stimuli provides an ideal tumor microenvironment. As an indication, this chronic inflammation has been shown to be a risk for some cancers and inflammation-induced atherosclerosis which later lead to acute cardiovascular diseases [68, 69]. Except for DGLA, diets having higher amounts of n-6 PUFAs are generally associated with inflammation (proinflammatory), constriction of blood vessels, and platelet aggregation [63]. DGLA has been considered a potent anti-inflammatory PUFA due to the oxygenated derivatives, series-1 PGs, particularly PGE1 and 15-hydroxyeicosatrienoic acid (15-HETrE) that both antagonize the synthesis of AA-derived proinflammatory eicosanoids [70]. Indeed, as it is well reviewed in Balić et al. [35], there is a lot of scientific and clinical evidence of the benefit of DGLA supplementation in the treatment and prevention of chronic inflammatory diseases like that of n-3 families, EPA and DHA. In particular, the combined DGLA and n-3 PUFA supplementation exhibits the highest diminishing inflammatory processes. Omega-3 PUFAs, such as EPA and DHA, on the other hand, have anti-inflammatory, antiaggregatory, vasodilation, and bronchodilation effects so that they help resolve inflammation and alter the function of vascular and carcinogen biomarkers, thus reducing the risk of cancer and CVD [71]. By considering these antagonistic effects of n-3 and n-6 PUFAs, both the n-3 and n-6 SC-PUFAs and LC-PUFAs in their absolute contents (as recommended by different expert bodies and organization) and in their proportional ratio with each other play a significant role in regulating body homeostasis of proinflammation and anti-inflammation, vasodilation and vasoconstriction, bronchoconstriction and bronchodilation, and platelet aggregation and antiaggregation [62].

## 8. Higher n-6/n-3 Ratio of Westernized Diets and the Modern Diet-Related Chronic Diseases

Compared with the Paleolithic diet in which human beings are evolved and their genetic patterns were established, westernized diets are deficient in n-3 PUFAs and have excessive amounts of n-6 PUFAs [16]. Excessive amounts of n-6 PUFAs or a high n-6/n-3 ratio has been shown to promote the pathogenesis of many diseases, including cardiovascular disease, cancer, and inflammatory and autoimmune diseases. As an indication, in India, by 2008, where the n-6/n-3 ratio was 38-50/1 at that time [5], the increased risks of modern diet-related chronic diseases have been associated with this high n-6/n-3 ratio [18, 72]. On the contrary, an increased level of n-3 PUFAs or a lower n-6/n-3 ratio with the ranges of 1-5/1 has been shown to employ suppressive effects depending on the diseases under consideration [5, 16]. A ratio of 4/1 was associated with a 70% decrease in total mortality from coronary heart disease. A ratio of 2.5/1 reduced rectal cell proliferation in patients with colorectal cancer, whereas a ratio of 4/1 showed a neutral effect. A ratio of 2-3/1 suppressed inflammation in patients with rheumatoid arthritis (RA), and a ratio of 5/1 had a beneficial effect on patients with asthma, whereas a ratio of 10/1 had adverse consequences [5, 18, 52]. Moreover, a 1/1 ratio was associated with the lower atherosclerotic formation in mice, and the severity of atherosclerosis increased as the n-6/n-3 ratio increases from 4/1 to 20/1 [73]. Indeed, as is well summarized by Simonetto et al. [74], Omega-3 PUFAs have a novel anti-inflammatory role in the prevention and treatment of inflammation in atherosclerosis and vascular cognitive impairment and dementia. Supplementation of Omega-3 PUFAs reduces the inflammatory pathway in atherosclerosis through the production of proinflammatory eicosanoids as well as by increasing the specialized proresolving mediators such as resolvins, protectins, and maresins. A ratio of 3/1 reduced the release of proinflammatory cytokine IL-6 on human patients with metabolic syndrome (inflammatory response) for a high-fat meal [75, 76]. Indeed, it seems that for this reason, both the ancient and modern hunter-gatherers following Paleolithic diets with an n-6/n-3 ratio of 1-2/1 were free of modern inflammatory diseases, like heart diseases, cancer, and diabetes, in which they are the primary cause of death and morbidity today [54]. This is now supported by the Mediterranean and Indo-Mediterranean diet styles with 4/1, which have shown a 70% reduction in mortality from coronary heart disease, possibly due to the low n-6/n-3 ratio [5, 16]. These experimental Mediterranean diet styles have a balanced n-6/n-3 ratio which is closest to Paleolithic diets but far from the ratio of westernized diet styles [72].

Western diets are the primary risk factors of modern diet-related chronic diseases by causing persistent chronic inflammation [77, 78]. Chronic diseases including obesity, type 2 diabetes, cardiovascular disease, cancer, and Alzheimer's disease are rising exponentially in the modern world [18, 79]. Though these diseases are multifactorial in nature, their prevalence is mostly associated with the unbalanced increase in dietary n-6 PUFAs and decrease in n-3 PUFAs in today's westernized diets, provided that there is opposing effect of n-6 and n-3 PUFAs on the development and suppression (prevention) of chronic diseases, respectively. Mostly, these diseases escalate on the fact that inflammation in conjunction with obesity is the basis of every chronic disease. The increased n-6/n-3 ratio in today's westernized diets has shown a rapid increase in the primary risk factors (predictor) for cardiovascular diseases such as obesity [5, 19]. On the contrary, maintaining the low ratio of n-6/n-3 has been concluded as a good strategy in the prevention and management of obesity [15, 16, 19].

The overconsumption of n-6 PUFAs than n-3 PUFAs could increase the production of AA-derived proinflammatory eicosanoids such as series-2 prostaglandins and thromboxanes and series-4 leukotrienes [5] as well as AA-derived ethanolamide and 2-acylglycerol endocannabinoids: arachidonoylethanolamine (AEA) and 2-arachidonoylglycerol (2-AG), respectively [16, 19], in our body which may alter the effects of these signals on their target cells. These eicosanoids and endocannabinoids act as intercellular messengers and mediators of inflammation, hyperactivity, cell proliferation, and immune reactivity. As eicosanoids from AA are very active to exert an effect even in very small quantities, if they are produced in much quantities, they cause the formation of thrombus and atheroma, the allergic reaction and inflammatory disorders, and the overmultiplication of cells (e.g., proliferation of adipocytes in adipose tissue) [5], whereas the endocannabinoids contribute to the hyperactive endocannabinoid system which leads into increased appetite and food intake [16, 19]. Recently, the role of AA-derived AEA and 2-AG endocannabinoids has been indicated to activate the endogenous cannabinoid receptors in the brain and resulted in increased appetite and food intake, leading to weight gain/obesity. In fact, obesity was formerly known due to the eicosanoids from AA such as PGI2 and PGF2*α* which activate the proliferation of white adipose tissue, decreasing its browning, respectively, while metabolites from EPA increase the browning of the adipose tissue, mitochondrial biogenesis, and thermogenesis [16]. The increased consumption of diets containing much amount of n-6 PUFAs is shown to change the normal physiological state to one that is prothrombotic and proaggregatory, with increased blood viscosity, vasospasm, and vasoconstriction and decreased bleeding time [24] and energy imbalances [16, 19].

AA-derived eicosanoids (prostaglandins, thromboxanes, leukotrienes, hydroxyl fatty acids, and lipoxins) [23, 80] as well as AA-derived AEA and 2-AG endocannabinoids [16, 19] are produced in larger quantities than those from n-3 PUFAs, especially EPA and DHA. Since AA is found in abundance in various cells and tissue including serum phospholipid, they can be readily converted into proinflammatory eicosanoids, endocannabinoids, and other products associated with inflammatory processes and chronic disorders in contrast to EPA and DHA [5, 16]. The increased ratio of AA to EPA+DHA in cells causes the increased production of reactive oxygen species, thereby oxidative stress in the cells [18]. Chronic inflammation may be involved in the pathogenesis of insulin resistance and type 2 diabetes mellitus, in which oxidative stress is the primary cause. Oxidative stress and inflammation can lead to subsequent insulin resistance, which places an increased risk for other chronic diseases. Free fatty acids and glucose induce inflammation via oxidative stress and have a cumulative independent effect which could be prevented by taking diets rich in n-3 PUFAs [72].

As it is well indicated in Valenzuela and Videla [81], chronic diseases such as nonalcoholic fatty liver disease (NAFLD) in obese individuals are commonly triggered by the increased oxidative stress in the liver. Oxidative stress in the liver is due to the higher accumulation and oxidation of fatty acids, insulin resistance, n-3 LC-PUFA depletion, and increased ratio of n-6/n-3 PUFAs which all favors the proinflammatory state. In the obese NAFLD (hepatic steatosis) patients, depletion or lower synthesis of n-3 LC-PUFAs (such as EPA and DHA) and insulin resistance with the simultaneous increment in the n-6/n-3 PUFA ratio have been observed [81, 82]. Depletion of n-3 LC-PUFAs may be caused by the following three mechanisms: (1) higher hepatic n-3 LC peroxidation and high susceptibility to free radical decomposition [81]; (2) obese patients with insulin resistance and oxidative stress and lower conversion of ALA to EPA and DHA by D-6-desaturase compared to LA to ARA, where D-6-desaturase is inversely associated with IR and oxidative stress because D-6-desaturase is upregulated by insulin and downregulated by LC-PUFAs such as in the presence of a higher ratio of ARA compared to EPA+DHA [81–83]; and (3) higher consumption of the powerful D-6-desaturase inhibitors such as *trans*-FAs (i.e., elaidic acid, 18:1n-9 *trans*) with lower consumption of EPA, DHA, and ARA [82]. On the contrary, in the study done by Valenzuela and Videla [84], the consumption of n-3 PUFAs in high quantities such as via supplements allows reducing hepatic steatosis as long as it is supplemented with antioxidants. This contributes by resolving the oxidative stress and inflammatory response in different tissues. In this study, EPA and DHA and extra virgin olive oil (EVOO) or EPA plus hydroxytyrosol attained a 66-83% reduction in high-fat diet-induced steatosis with the simultaneous inhibition of the proinflammatory state associated with steatosis. These supplementations come to effect by triggering different molecular mechanisms that modify antioxidant, antisteatotic, and anti-inflammatory responses [84, 85].

Higher intake of n-6 PUFAs such as LA has been observed to generate lower n-3 LC-PUFAs (EPA and DHA) in obesity during pregnancy and breastfeeding [86]. With this increased LA consumption, the DHA concentration in erythrocytes and breast milk was significantly reduced in the pregnancy and lactation periods but not for AA. Increased n-6 fatty acids in diets disrupt the balance of pro- and anti-inflammatory agents in the body, promoting chronic inflammation which elevates the risk of cardiovascular diseases, cancer, and other chronic diseases. The higher the ratio of n-6/n-3 PUFAs in platelet PLs, the higher the death rate from cardiovascular diseases and type 2 diabetes mellitus due to the higher production of proinflammatory agents in the body [87]. In contrast, a lower ratio of n-6/n-3 PUFAs is desirable in reducing the risk of many of the chronic diseases [15, 76, 88–90].

Overconsumption of dietary n-6 PUFAs, coupled with low intake of n-3 PUFAs, is identified in the larger production of biomarkers, such as thromboxane A2 (TXA2), leukotriene, prostacyclin, interleukin-1 and interleukin-6, tumor necrosis factor-*α*, and C-reactive proteins (CRPs), and oxidative stress, with adverse proinflammatory effects, resulting in diet-related chronic diseases such as cardiovascular diseases, diabetes mellitus, obesity, some cancers, autoimmune diseases, rheumatic arthritis, asthma, and depression which are associated with the increased production of cell signaling molecules in the tissues such as thromboxane A2 (TXA2), leukotriene, prostacyclin, interleukin-1 and interleukin-6, tumor necrosis factor-*α*, and C-reactive proteins [18, 47]. When the ratio between the n-6 and n-3 PUFAs is balanced, the gene expression, lipid mediator metabolism, and cytokine production are performed in their normal physiological state [18]. In different studies, the optimum physiologic ratio of n-6/n-3 was investigated using human and animal models which have a specific disease as indicated in [73–76]. In most of these studies, the n-6/n-3 ratio that has been shown to employ oppressive effects on the disease under consideration was found between the ranges of 4-5/1. Hence, ratios of 4-5/1 or less are highly recommended and are considered the optimum dietary intake ratio [33, 56]. On the other hand, diets especially with greater than a 10/1 ratio are not recommendable [33].

## 9. Conclusion

Though both n-3 PUFAs and n-6 PUFAs are generally considered to have beneficial health effects, they have antagonistic (opposing) metabolic functions in the body which later lead to pathological processes in the human body. The n-6 PUFAs largely exceed the amount of n-3 PUFAs in most westernized diets. Due to this higher n-6 consumption than n-3 PUFAs, modern diet-related chronic diseases have increased from time to time following the increasing unbalanced consumption ratio between these two families. In addition to the higher ratio of n-6/n-3, the essential fatty acids of LA and ALA compete for the same set of enzymes on the elongation and desaturation processes into longer PUFAs. Longer PUFAs had also undergone competitive oxidative metabolism for the same set of enzymes. The LA PUFAs which are in a higher proportion than ALA were converted in a higher percentage of AA and other n-6 PUFAs; hence, most of the cellular PLs are occupied by AA. On oxidative metabolism, AA produces much potent proinflammatory eicosanoids and they contribute to the formation of thrombus and atheroma, to the allergic reaction and inflammatory disorders, and to the overmultiplication of cells as well as to the hyperactive endocannabinoid system in which appetite and food intake which can lead to weight gain/obesity are increased. Omega-3 PUFAs, such as EPA and DHA, on the other hand, have anti-inflammatory, antiaggregatory, vasodilation, and bronchodilation effects so that they help resolve inflammation and alter the function of vascular and carcinogen biomarkers, thus reducing the risk of cancer and cardiovascular diseases. Therefore, by considering these antagonistic effects of n-3 and n-6 PUFAs, both the n-3 and n-6 SC-PUFAs and LC-PUFAs in their proportional ratio with each other, which is as close as 4-5/1, play a significant role in regulating body homeostasis of inflammation and anti-inflammation, vasodilation and vasoconstriction, bronchoconstriction and bronchodilation, and platelet aggregation and antiaggregation.

## Figures and Tables

**Table 1:** List of the most common n-3 and n-6 PUFAs. Data elaborated from Ruiz-Lopez et al. [27] and Russo [33].

Omega family	Common name	Systematic name	n and *Δ* abbreviations
n-3	*α*-Linolenic acid (ALA)	*all*-*cis*-9,12,15-Octadecatrienoic acid	18:3n-3 or 18:3^*Δ* 9,12,15^
	Stearidonic acid (SDA)	*all*-*cis*-6,9,12,15-Octadecatetraenoic acid	18:4n-3 or 18:4^*Δ*6,9,12,15^
	Eicosatetraenoic acid (ETA)	*all*-*cis*-8,11,14,17-Eicosatetraenoic acid	20:4n-3 or 20:4^*Δ*8,11,14,17^
	Eicosapentaenoic acid (EPA)	*all*-*cis*-5,8,11,14,17-Eicosapentaenoic acid	20:5n-3 or 20:5^*Δ*5,8,11,14,17^
	Clupanodonic acid (DPA_n-3_)	*all*-*cis*-7,10,13,16,19-Docosapentaenoic acid	22:5n-3 or 22:5^*Δ*7,10,13,16,19^
	Tetracosapentaenoic acid (TPA)	*all*-*cis*-9,12,15,18,21-Tetracosapentaenoic acid	24:5n-3 or 24:3^*Δ*9,12,15,18,21^
	Nisinic acid (THA)	*all*-*cis*-6,9,12,15,18,21-Tetracosahexaenoic acid	24:6n-3 or 24:6^*Δ*6,9,12,15,18,21^
	Docosahexaenoic acid (DHA)	*all*-*cis*-4,7,10,13,16,19-Docosahexaenoic acid	22:6n-3 or 22:6^*Δ*4,7,10,13,16,19^

n-6	Linoleic acid (LA)	*all*-*cis*-9,12-Octadecadienoic acid	18:2n-6 or 18:2^*Δ*9,12^
	*γ*-Linolenic acid (GLA)	*all*-*cis*-6,9,12-Octadecatrienoic acid	18:3n-6 or 18:3^*Δ*6,9,12^
	Dihomo-*γ*-linolenic acid (DGLA)	*all*-*cis*-8,11,14-Eicosatrienoic acid	20:3n-6 or 20:3^*Δ*8,11,14^
	Arachidonic acid (AA)	*all*-*cis*-5,8,11,14-Eicosatetraenoic acid	20:4n-6 or 20:4^*Δ*5,8,11,14^
	Adrenic acid (DTA)	*all*-*cis*-7,10,13,16-Docosatetraenoic acid	22:4n-6 or 22:4^*Δ* 7,10,13,16^
	Tetracosatetraenoic acid (TTA_n-6_)	*all*-*cis*-9,12,15,18-Tetracosatetraenoic acid	24:4n-6 or 24:4^*Δ*9,12,15,18^
	Tetracosapentaenoic acid (TPA_n-6_)	*all*-*cis*-6,9,12,15,18-Tetracosapentaenoic acid	24:5n-6 or 24:6^*Δ*6,9,12,15,18^
	Osbond acid (DPA_n-6_)	*all*-*cis*-4,7,10,13,16-Docosapentaenoic acid	22:5n-6 or 22:5^*Δ*4,7,10,13,16^

**Table 2:** n-3 and n-6 PUFA composition of predominant fatty acid dietary sources (vegetable oils, oily seed crops, fish, and microalgae). Data elaborated from Saini and Keum [12], Balić et al. [35], and Amjad et al. [51].

Sources of dietary PUFAs		n-3 (g)			n-6 (g)			Ref.
		ALA	EPA	DHA	LA	AA	DPA_n-6_	
Oils (purified)	Flaxseed (oil)	53.37	—	—	14.33	—	—	[12, 35]
	Canola (oil)	9.15	—	—	18.65	—	—	
	Soybean (oil)	7.6	—	—	51.36	—	—	
	Wheat germ (oil)	5.3	—	—	55.1	—	—	
	Corn (oil)	0.6	—	—	49.83	—	—	
	Sunflower (oil)	0.33	—	—	49.89	—	—	
	Safflower (oil)	0.1	—	—	12.72	—	—	

Seed and nuts	Chia (dried/ground)	17.83	—	—	5.84	—	—	[12, 35]
	Walnut (dried/ground)	6.64	—	—	34.02	—	—	
	Hazelnut (dried/ground)	0.11	—	—	5.09	—	—	
	Almond (dried/ground)	0.3	—	—	10.54	—	—	
	Hemp seed (hulled)	8.68	—	—	27.36	—	—	
	Brazil nuts (dried)	0.02	—	—	23.83	—	—	

Vegetables	Lettuce (raw)	0.15	—	—	0.06	—	—	[35]
	Green broccoli (raw)	0.11	—	—	0.03	—	—	
	Brussels (raw)	0.17	—	—	0.08	—	—	

Fish (oil)	Salmon (oil)	—	13.3	18.23	—	—	2.99	
	Sardine (oil)	—	10.15	10.66	—	—	1.97	
	Herring (oil)	—	6.28	4.21	—	—	0.62	
	Menhaden (oil)	—	13.18	8.56	—	—	4.92	[12, 35]

Fish (raw)	Salmon (raw)	0.09	0.89	1.19	0.15	0.05	—	
	Herring (raw)	0.19	1.09	1.01	0.22	0.1	—	
	Sardine (raw)	—	0.51	1.16	0.06	0.04	—	
	Trout (raw)	0.1	0.15	0.5	0.37	0.05	—	
	Cod (dried)	—	0.02	0.62	0.03	0.12	—	[35]

Beef	New Zealand, kidney (cooked)	0.08	0.15	0.03	0.38	0.37	0.10	
	New Zealand, liver (raw)	0.05	0.11	0.04	0.14	0.19	0.14	
Lamb	New Zealand, brain (raw)	0.00	0.00	0.36	0.01	0.16	0.14	[12]

Microalgal (oil)	*Phaeodactylum tricornutum*	—	36.5	23.6	—	—	—	
	*Chlorella minutissima*	—	29	—	—	—	—	
	*Nannochloropsis oceanica*	—	23.4	—	—	—	—	
	*Nannochloropsis salina*	—	28	—	—	—	—	
	*Crypthecodinium cohnii*	—	—	41	—	—	—	
	*Ceratium horridum*	—	—	29.3	—	—	—	[51]

Values are g/100 g of total fatty acids.

**Table 3:** Fatty acid composition (%) and n-6/n-3 ratio of different vegetable oils, animal fat, fish oils, and microalgal oils.

Dietary fat		SFA (%)	MUFA (%)	n-3 PUFAs (%)	n-6 PUFAs (%)	n-6/n-3	Ref.
Vegetable oils	Sunflower oil	12	20.5	0.10	63.2	632/1	[51]
		12	16	1	71	71/1	[54]
	Corn oil	14.5	29.9	0.9	50.4	56/1	[51]
		13	29	1	57	57/1	[54]
	Soybean oil	15.6	21.2	7.3	51.5	7.05/1	[51]
		15	23	8	54	6.75/1	[54]
	Palm oil	47.8	37.1	0.3	10.1	33.66/1	[51]
		51	39	0	10	10/0	[54]
	Olive oil	14.3	73	0.7	7.8	11.14/1	[51]
		15	75	1	9	9/1	[54]
	Flaxseed oil	9	18	57	16	0.28/1	[54]
	Peanut oil	19	48	0	33	33/0	
	Cottonseed oil	27	19	0	54	54/0	
	Canola oil	7	61	11	21	1.91/1	
	Safflower oil	8	77	1	14	14/1	
	Coconut oil	91	7	0	2	2/0	

Animal fat	Butter	68	28	1	3	3/1	[54]
	Lard	43	47	1	9	9/1	

Fish oils	Cod liver	22.6	20.7	19.8	0.9	0.045/1	[51]
	Herring	21.3	56.6	11.9	12	1.01/1	
	Salmon	19.9	17.0	35.3	1.06	0.03/1	
	Sardine	30.4	14.5	28.1	2.2	0.078/1	

Microalgal oils	*Phaeodactylum tricornum*	19	33	20 (ALA+EPA+DHA)	7 (LA+AA)	0.35/1	[48]
	*Nannochloropsis gaditana*	15	35	44 (ALA+EPA)	4 (AA)	0.09/1	
	*Pavlova lutheri*	20	28	29 (ALA+EPA+DHA)	1 (LA)	0.03/1	
	*Ectocarpus siliculosus*	—	16	43 (ALA+EPA)	16 (LA+AA)	0.37/1	
	*Fucus vesiculosus*	21	28	15 (ALA+EPA)	25 (LA+AA)	1.66/1	
	*Chondrus crispus*	34	15	23 (ALA+EPA)	19 (LA+AA)	0.82/1	

SFA: saturated fatty acid; MUFA: monounsaturated fatty acid.
